# Nutritional, Nutraceutical, and Medicinal Potential of *Cantharellus cibarius* Fr.: A Comprehensive Review

**DOI:** 10.1002/fsn3.4641

**Published:** 2024-12-13

**Authors:** Ajay Kumar, Reema Devi, Rajni Dhalaria, Ashwani Tapwal, Rachna Verma, Summya Rashid, Gehan M. Elossaily, Khalid Ali Khan, Kow‐Tong Chen, Tarun Verma

**Affiliations:** ^1^ Forest Protection Division Shimla India; ^2^ Department of Biotechnology ASBASJS Memorial College Punjab India; ^3^ Department of Pharmacology & Toxicology, College of Pharmacy Prince Sattam Bin Abdulaziz University Al‐Kharj Saudi Arabia; ^4^ Department of Basic Medical Sciences, College of Medicine AlMaarefa University Riyadh Saudi Arabia; ^5^ Applied College, Center of Bee Research and its Products (CBRP), and Unit of Bee Research and Honey Production King Khalid University Abha Saudi Arabia; ^6^ Department of Occupational Medicine Tainan Municipal Hospital (Managed by ShowChwan Medical Care Corporation) Tainan Taiwan; ^7^ Department of Public Health, College of Medicine National Cheng Kung University Tainan Taiwan; ^8^ Department of Dairy Science and Food Technology, Institute of Agricultural Sciences Banaras Hindu University Varanasi India

**Keywords:** *Cantharellus cibarius*, culinary, ectomycorrhizal, medicinal, nutraceutical, nutritional

## Abstract

Mushrooms are considered as nutraceutical foods that can effectively prevent diseases such as cancer and other serious life‐threatening conditions include neurodegeneration, hypertension, diabetes, and hypercholesterolemia. The *Cantharellus cibarius*, also known as the “Golden chanterelle” or “Golden girolle,” is a significant wild edible ectomycorrhizal mushroom. It is renowned for its delicious, apricot‐like aroma and is highly valued in various culinary traditions worldwide. It is well known for its nutritional, nutraceutical, and therapeutic properties. The high nutritional value of *C. cibarius* is attributed to its abundant carbohydrates, proteins, β‐glucans, dietary fiber, and low‐fat content. It also contains medicinal polysaccharides (β‐glucans), proteins (lectins and selenoproteins), important fatty acids (linoleic and omega‐6), vitamins, and minerals (N, P, K, Ca, Zn, Ag, Se, etc.). The sporocarp of *C. cibarius* contains a diverse array of bioactive metabolites, including flavonoids, phenolics, sterols, fatty acids, organic acids, indole groups, carbohydrates, vitamins (tocopherols), amino acids, enzymes, bioelements, carotenoids, and 5ˊ‐nucleotides. *C. cibarius* has a wide array of biological properties, such as antioxidant, anticancer, anti‐inflammatory, antifungal, antibacterial, anthelmintic, insecticidal, antihypoxia, antihyperglycemic, wound‐healing, cytotoxic, and iron‐chelating activity. Thus, the present review gives an overview of *C. cibarius*, covering its chemical composition, ecological significance, postharvest preservation strategies, and potential applications in dietary supplements, nutraceuticals, and pharmaceuticals. It also dives into the etymology, taxonomy, and global distribution of the renowned “Golden Chanterelle.” Furthermore, there is a need to valorize waste materials created during production and processing, as well as to acquire a thorough understanding of the mechanisms of action of bioactive compounds in mushrooms.

## Graphical abstract

This review explores the nutritional, nutraceutical, and medicinal potential of Cantharellus cibarius, known as the “Golden chanterelle.” Emphasizing its rich composition of bioactive compounds and wide range of health benefits, the review also covers ecological significance, preservation strategies, and potential applications in dietary supplements, nutraceuticals, and pharmaceuticals. Additionally, it highlights the need for further research on waste valorization and the mechanisms of action of mushroom bioactive compounds.

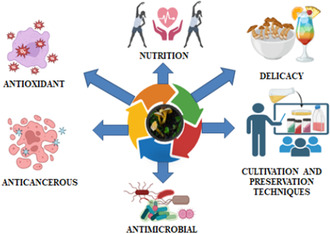

**Abbreviations**
CAD $: Canadian dollar
CCP: Cantharellus crude polysaccharide
CDK: Cyclin‐dependent kinase
DPPH: 2,2‐diphenyl‐1‐picrylhydrazyl
DW: Dry weight
FAOSTAT: Food and Agriculture Organization statistical
FIP: Fungal immunomodulatory proteins
FW: Fresh weight
hMNC: Human mononuclear cell
IL‐6: Interleukin‐6
iNOS: Inducible nitric oxide synthase
IU: International unit
kPa: Kilopascal
LOX: Lipoxygenase
MCF‐7: Michigan cancer foundation‐7
MFC: Minimum fungicidal concentration
MIC: Minimum inhibitory concentration
MRC‐5: Medical research council‐5
MUFA: Monounsaturated fatty acid
NFκB: Nuclear factor kappa‐light‐chain‐enhancer of activated B cells
NK: Natural killer
PAN‐C1: Pancreatic cancer cell line1
pRb: Phosphorylated retinoblastoma protein
PUFA: Polyunsaturated fatty acid
RIP: Ribosome‐inactivating proteins
ROS: Reactive oxygen species
SFA: Saturated fatty acid
TNF‐α: Tumor necrosis factor
TLR4: Toll‐like receptor 4
USD: United state dollar
USFA: Unsaturated fatty acid

## 1 Introduction

Fungi are the most diverse organisms on the glove containing second‐highest number of species after insects (Purvis and Hector 2000). Chytridiomycota, Zygomycota, Ascomycota, Basidiomycota, and Glomeromycota are the five primary phyla that make up the kingdom fungi (Arazo 2021). Based on their morphology and life cycles, fungi can be categorized into three main groups: single‐celled yeasts, multicellular filamentous molds, and macroscopic filamentous fungi (mushrooms) (Wakai et al. 2017). There are estimated 1.5 million species of fungi that can be grown in culture (Hawksworth 1991), but recent estimate accounts for 2.2–3.8 million of species worldwide (Hawksworth and Lücking 2017), while Wu et al. (2019) suggested an estimate of 11.7–13.2 million fungal species on the planet. The estimated record of the fungal fossil can be traced back to date as far as 900 million years with all major groups were present around 300 million years ago (Taylor, Remy, and Hass 1994).

There are estimated 150,000–160,000 different species of mushrooms, of which only around 14,000 have been identified (Chang and Wasser 2017). A macro‐fungus, or mushroom, is mainly restricted to the phylum Basidiomycota and Ascomycota of the kingdom fungi (Rathore et al. 2019; Singh and Passari 2018). Mushroom possesses distinctive sporocarp with a pileus that is supported by a stipe, thin membranous vellum that is produced during the development, and lamellae that form basidiospores (Mau, Miklus, and Beelman 1993; Shen et al. 2017) or ascospores. The sporocarp can be seen with naked eyes and grow either epigeous or hypogeous (Niazi and Ghafoor 2021). Majority of mushrooms are the members of phylum Basidiomycota, which includes about 35,000 different species (He et al. 2019). Of the 16,000 identified mushrooms, approximately 7000 exhibit varying levels of edibility (Hawksworth 2012). Primarily, there are about 3000 species of edible mushrooms, and approximately 700 have therapeutic potential (Wasser 2002; Kalac 2013; Chang and Wasser 2017; Li et al. 2021). Around 350 different types of mushrooms are consumed by people worldwide (Willis 2018). Mushrooms and fungi have more than 100 medicinal functions, and the key medicinal properties include antioxidant, antifungal, antibacterial, antiviral, antiparasitic, anticancer, antidiabetic, anti‐inflammatory, anti‐allergic, immuno‐modulating, cardiovascular protector, anticholesterolemic, hepatoprotective, and detoxification effects; they also guard against tumorigenesis processes (Lindequist, Niedermeyer, and Jülich 2005; Zhang et al. 2011; Chang and Wasser 2012; Ruthes, Smiderle, and Iacomini 2016). Mushrooms possess chemically diverse secondary metabolites that exhibit several types of biological functions which are evaluated in both conventional medicine and new targets of molecular biology (Khatun et al. 2012).

*Cantharellus*, *Craterellus*, *Polyozellus*, and *Gomphus* are the four genera that are usually referred to as “Chanterelles,” on the basis of similar appearance of their spore‐bearing surfaces without magnification (Pilz et al. 2003). On the basis of multilocus phylogenetic analyses, *Cantharellus* species have been characterized into seven subgenera (Buyck et al. 2014). There are currently about 300 of *Cantharellus* species identified worldwide (Cao et al. 2021). *Cantharellus* species are widely dispersed and particularly abundant in subtropical to tropical regions (Corner 1966; Buyck et al. 2013, 2014) and are being harvested since centuries from Europe, North America, Africa, and Asian forests, and on every continent, it is associated with the suitable host tree. The bicyclic carotenoid pigments and octenols attributing the characteristic odor of these mushrooms (Arpin and Fiasson 1971; Pyysalo 1976; Gill and Steglich 1987), thus due to their excellent flavor and apricot‐like aroma (Foltz, Perez, and Volk 2013).

*C. cibarius* Fr. is globally known as “Chanterelle” or “Golden Chanterelle” and also recognized as golden girolle. The most well‐known and widely consumed edible species in the *Cantharellus* genus is *Cantharellus cibarius*. It grows widely from June to October in India, Thailand, China, Africa, and America, and in several other European countries (Boa 2004; Eyssartier et al. 2009; Olariaga et al. 2017; Bulam, Ustun, and Peksen 2021). It is used as natural ingredient in traditional European and Asian medicines in curing liver, lungs and stomach diseases, spleen, and eyesight problems (Bulam, Ustun, and Peksen 2021). *C. cibarius* is known to exhibit antimicrobial (Santoyo et al. 2009; Aina et al. 2012; Novakovic et al. 2019; Chen and Xu 2024), insecticidal (Mier et al. 1996; Cieniecka‐Rosłonkiewicz et al. 2007), scavenging of lipid peroxidation (Palacios et al. 2011), antiaging, pain killer, antioxidant, and anticancer properties (Ebrahimzadeh et al. 2010; Muszyńska, Sułkowska‐Ziaja, and Ekiert 2011; Zaidman et al. 2005; Chen and Xu 2024).

The red list has classified *Cantharellus* and *Craterellus* as endangered species in many European nations (Arnolds 1995; Larsson 1997). In Netherland, the population of *C. cibarius* has been decreased by 60% between 1960 and 1980 (Jansen and Van Dobben 1987; Arnolds 1995). The removal of wood logs and other water‐holding substances from forests (Norvell 1992a, 1992b; Molina et al. 1993) might also result in a reduction in the formation of carpophores. Researchers have paid a lot of interest lately on the bioactive substances obtained from various mushroom species and their potential for use in dietary supplements and pharmaceuticals. This paper provides an overview of current understanding regarding chemical make‐up, ecology, postharvest preservation methods, and prospective use in dietary supplements, nutraceuticals, and pharmaceuticals in reference to *C. cibarius*.

### 1.1 Methodology

A detailed compilation of information was created from published sources, covering the scientific name, family, distribution, nutritional, nutraceutical, culinary, and preservation aspects of this mushroom species. Various key phrases were employed to search for articles in online databases such as Science Direct, PubMed, Google Scholar, and Web of Science, using terms like *Cantharellus cibarius*, ectomycorrhizal, nutritional, nutraceutical, medicinal, and culinary. This study analyzed a range of scientific literature, including review articles, research papers, and books published up to 2024.

## 2 Etymology and Taxonomy

In reference to their funnel‐like shape, the “Chanterelle” word is taken from a Greek term “kantharos,” meaning “cup,” “goblet,” or “drinking vessel” (Persson and Mossberg 1997). ″Chantarellen″ were recognized as common edible mushrooms by a Swedish naturalist Linnaeus (1747). Later he termed the golden chanterelle with the scientific name *Agaricus chantarellus* (Linnaeus 1755). It was Elias Fries, a Swedish scientist and botanist, who first time describes and assigned the current scientific name ″*Cantharellus cibarius″* for golden chanterelle in his book Systema Mycologicum (Fries 1821), and later in 1905, the species was typified with *Cantharellus cibarius* Fr. (Earle 1905). *Cantharellus* was initially described as an artificial assembly of species with veins or folds similar to hymenophore by early authors (Fries 1821; Fries 1874; Fuckel 1870; Quélet 1888), later that broad idea was subsequently narrowed to species in the cantharelloid clade (Moncalvo et al. 2006; Buyck et al. 2014). The family Cantharellaceae was later revised by Petersen (1971a, 1971b) and Romagnesi (1995) using classical microscopic and chemical characteristics.

One of the eight major clades of homobasidiomycetes is the cantharelloid clade, having polyphyletic origin and includes the genus *Cantharellus* as a key member (Hibbett and Thorn 2001; Pilz et al. 2003; Binder et al. 2005; Moncalvo et al. 2006). The species in genus *Cantharellus* are characterized by gymnocarpic sporocarps that are fleshy, terricolous, and enduring, but not perennial (Smith and Morse 1947; Petersen 1985; Danell 1994a; Pegler, Roberts, and Spooner 1997; Cairney and Chambers 1999). Specific epithet “*cibarius”* is a Latin word which means “food or nutriment” (Lewis 1891). *C. cibarius* has a medium to big fruiting body that is generally egg‐yolk yellow in color. Gills that extend down the stalk are blunt, thick, branched, and widely spaced. At first, the cap is shallowly convex, but it quickly becomes flat, and at the center, it depresses shallowly to deeply. The margin of cap is frequently wavy and indented, and it can range from a bright yellow to an orange‐yellow that darkens when bruised, but can also fade to whitish in sunshine. Fruit body smells vaguely of dried apricots or is absent, and flavor is unremarkable. Stalk is solid, tapers downward from cap. Spore print has a light‐yellow color. Spores are smooth, ellipsoid, thin‐walled, and measure 8–11 × 4.5–5.5 μm (McKnight and McKnight 1987). Hyphae are monomitic; cystidia are absent and clamp connections are present.

## 3 Ecology and Distribution

**FIGURE 1:**
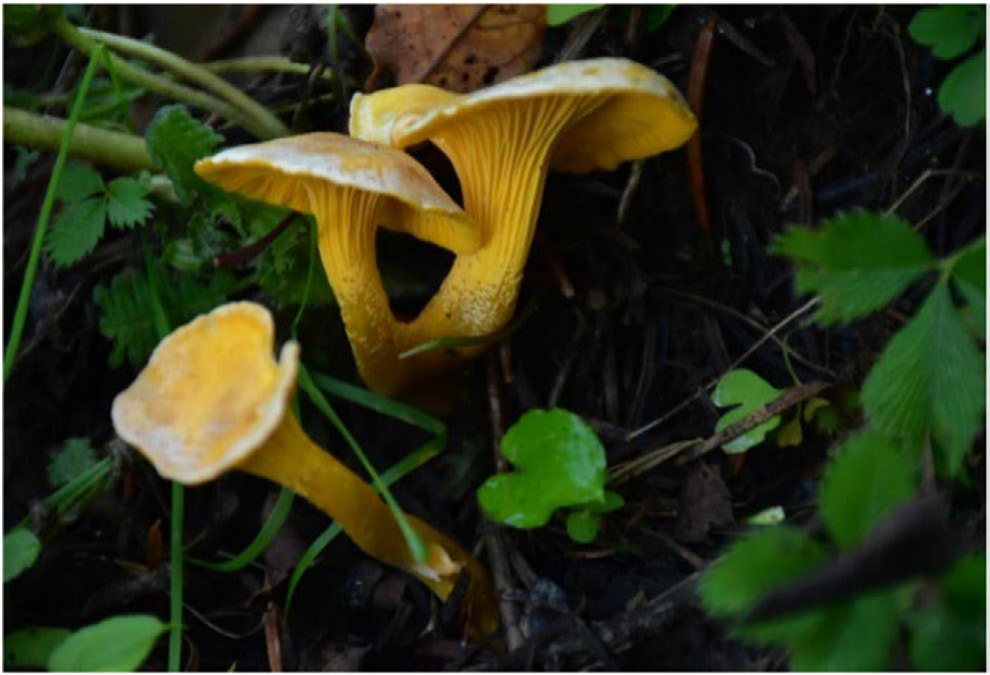
Photograph showing *C. cibarius* in its natural habitat.

*C. cibarius* as shown in Figure 1 is the wellspring of the majority of information about the ecology of species in the *Cantharellus* genus (Danell 1994a). Ecological preferences of *C. cibarius* estimated with Ellenberg's scales indicate that the species prefers a temperate climate, shade‐resistant, and is rarely observed in full illumination; mesophyte, as determined by the soil humidity scale and prefer acidic nitrogen‐poor soils, with neutral nitrogen‐rich soils serving as an exception (Luginina and Sorokina 2021).

The species of *Cantharellus* genus reacts to primary soil factors like pH, organic matter, and drainage (Jansen and Van Dobben 1987), as well as secondary factors, for instance, humus properties (Nantel and Neumann 1992) and nitrogen (Wallenda and Kottke 1998). *C. cibarius* tends to favor mostly sandy soils (Jansen and Van Dobben 1987), with low nitrogen content and a pH range of 4.0–5.5 (Jansen and Van Dobben 1987; Danell 1994a; Rangel‐Castro 2001). Besides that, other important variables have been investigated and confirmed to be crucial for the fructification of chanterelles, such as needle cover and duff depth (Bergemann and Largent 2000), moisture, vascular plants, air and soil temperature (Amaranthus and Russell 1996), and canopy coat (Pilz, Molina, and Mayo 2006). Throughout the world, the extensive host range of *C. cibarius* includes coniferous and broad‐leaved plants. However, certain physiological strains are specifically adapted to particular host trees. In boreal woodlands, chanterelle sporocarps first appear in mid‐July and continue throughout late October (Danell, 1994a, 1994b), and its development might differ significantly on locations and yearly basis (Egli et al. 2006). In southern taiga and subtaiga zones, the species is primarily found in pure pine forests or mixed (spruce and birch) in the tree stands of lichen, green‐moss and cowberry types, with low to medium crown densities (Luginina and Sorokina 2021). Fruit bodies of *C. cibarius* are generally found in the association with older trees (Danell 1994a, 1994b). Moreover, the temperature and host's growth rate figure out the golden chanterelles production in forests when the trees are between 10 and 40 years old (Okan et al. 2014).

*Cantharellus* is an ectomycorrhizal genus, forms mutualistic relationship with several economically valuable trees (Redhead, Norvell, and Danell 1997), and includes members of Pinaceae, Salicaceae, Betulaceae, Fagaceae, Juglandaceae, and Leguminosae (Danell 1999; Kumari, Upadhyay, and Reddy 2011; Buyck et al. 2014; Henkel et al. 2014; De Kesel et al. 2016; Ogawa et al. 2018; Cao et al. 2021; Shao et al. 2021). The genus *Cantharellus* includes about 23 species in North America, nine in Europe, seven in each of South America and Australia, 46 in Africa, 3 in New Zealand, and 19 in Asia (Eyssartier 2003; Tibuhwa et al. 2008; Eyssartier et al. 2009; Buyck and Hofstetter 2011; Buyck et al. 2011; Shao, Tian, and Liu 2011).

## 4 Nutritional Profiles

Mycophagy is a practice of eating mushrooms. The custom of collecting and eating wild edible mushrooms has a long history (Ho, Zulkifli, and Tan 2020). According to their intended application, mushrooms can be conveniently classified into the following three categories: edible (54%), wild (8%), and medicinal (38%) (Royse, Baars, and Tan 2017). A “Mushroom” could be poisonous, edible, or unpleasant because the distinction between poisonous and edible fungi is not always discernible (Hay 1887; Arora 1986). Mushrooms are excellent source of amino acids, proteins, glycogen, lipids, vitamins (Okhuoya et al. 2010), and minerals like phosphorus, magnesium, selenium, copper, and potassium (Mallikarjuna et al. 2013), fibers, phenolic compounds (Bano and Rajarathnam 1988; Lu et al. 2020), chitin, and β‐glucans (Feeney et al. 2014). High selenium content (63 μg/g DW) was reported in *C. cibarius* (Kolundzic et al. 2017), and selenium is an important element of rare amino acid (selenocysteine), that constitute selenoprotein. The selenoprotein plays a vital role as an antioxidant and hence reduces heart diseases and boosts immunity and cancer chemoprevention (Tinggi 2008). Selenium and copper both possess antioxidant properties that protect against damaging free radicals, therefore implicated as potential anticarcinogenic agents (Satyanarayana et al. 2006; Zuo et al. 2006).

Since the Medieval era, Chanterelles (*Cantharellus* spp.) have been recognized as among the most beloved edible mushrooms (Lobelius 1581; Danell 1994a). *Cantharellus* mushrooms are well recognized and are a preferred cuisine in various parts of the world. This mushroom is widely appreciated for its fruity, apricot‐like aroma. *C. cibarius* is abundant in proteins, carbohydrates, vitamins, minerals, and aromatic compounds (Bak et al. 2023; Chen and Xu 2024; Senila, Senila, and Resz 2024). High nutritional value of *C. cibarius* is due to its high amount of carbohydrates (31.9% DW), proteins (53.7% DW), β‐glucans, dietary fiber, and low levels of fat content (2.9% DW) (Muszyńska et al. 2016). Linoleic (654.706 mg kg^−1^ DW) and oleic acids (148.168 mg kg^−1^ DW) represent the majority of its fatty acids (Bulam, Ustun, and Peksen 2021). Egwim, Elem, and Egwuche (2011) determined proximate composition of edible wild mushrooms including *C. cibarius* and reported carbohydrate (18%), crude protein (26.25%), fat (9.14%), and crude fiber (13.64%). Kolundzic et al. (2017) reported α‐glucan, β‐glucan, and total glucans in dry, cooked as well as in methanolic and aqueous extract of *C. cibarius*. The amount of β‐glucans was 14.90–0.38 mg/g in a dried sample. It possesses the highest quantity of threonine and lysine as 8.98 and 5.74 mg g^−1^ DW, respectively (Beluhan and Ranogajec 2011; Muszyńska et al. 2016; Nyman et al. 2016). It contains several vitamins, notably thiamin, riboflavin, pantothenic acid, niacin, and ascorbic acid. Additionally, it also contains the vitamin B complex, vitamin A, E, D2, and minerals including calcium (973.17), potassium (67411.93), and phosphorus (5126.47), with low amount of selenium as 0.61 and 0.153 mg kg^−1^ DW (Costa‐Silva et al. 2011; Muszyńska et al. 2016; Bulam, Pekşen, and Ustun 2019; Bulam, Ustun, and Peksen 2019).

### 4.1 Carbohydrates

Mushrooms are a promising source of novel prebiotic components since they are rich in indigestible carbohydrates (Sawangwan et al. 2018; Aida et al. 2009). Moreover, there is belief that mushrooms having high mannitol content and lower glycemic index are beneficial for diabetic persons (Kozarski et al. 2015). Additionally, mushrooms also contain a high amount of complex polysaccharides. These compounds are pharmacologically crucial, and for example, lectins have antitumor and immunomodulating attributes (Wang et al. 1996; Imberty et al. 2000; Yau et al. 2015), hypotensive effects (Tam et al. 1986), and antiangiogenesis effects (Jana and Acharya 2020). The common carbohydrates of various mushrooms that display significant biological activities are fructose, glucose, xylose, mannose, fucose, rhamnose, maltose, mannitol, sucrose, and trehalose (Zaidman et al. 2005; Zhang et al. 2007; Ferreira, Barros, and Abreu 2009; Alves, Ferreira, Dias, et al. 2013; Alves, Ferreira, Froufe, et al. 2013).

In *C. cibarius*, carbohydrates comprise 31.9% (Muszyńska et al. 2016) and 66.07 g/100 g DW (Ouzouni et al. 2009). Total carbohydrate content in *Craterellus cibarius* was 12.25 and 58.67 mg g^−1^ in fresh and dry weight, respectively; 33.63 and 49.99 mg g^−1^ in fresh and dry weight of *Cantharellus tubiformis*, respectively; and 62.75 and 144.38 mg g^−1^ in fresh and dry weight of *C. cibarius*, respectively (Salihovic et al. 2021). Chanterelle contains trehalose (6.68 g), mannitol (8.56 g), mannose (8.56 g), and glucose (7.98 g) per 100 g dry weight (Kumari, Reddy, and Upadhyay 2011). In reference to fresh weight, Croatian *C. cibarius* contains 31.91 g carbohydrates, 2.9 g of lipids, 8.8 g of ash, and 118 kJ of energy (Muszyńska et al. 2016). *C. cibarius* harvested from Greece contain more sugar contents (66.07 g/100 g DW) as compared to croatian counterpart (Ouzouni et al. 2009; Beluhan and Ranogajec 2011). In *Cantharellus* spp., high carbohydrate concentration (64.24%) was observed by Ugbogu et al. (2020), approximately 54% for *C. cibarius* (Panchak et al. 2020), and 30.4 mg g^−1^DW of extract in *C. cibarius* (Kozarski et al. 2015). Two glucan type polysaccharides (PsCcib‐I and II) were isolated from the wild edible *C. cibarius*. The polysaccharide isolated from hot aqueous NaOH fraction (PsCcib‐II) exhibited a triple helical conformation (Villares et al. 2013). Polysaccharides of chanterelles like other carbohydrates (β‐glucans) of Basidiomycota species exhibited multifrontal antioxidant, anticancer, or chemoprevention activities (Jin and Lu 2011; Nowacka‐Jechalke, Olech, and Nowak 2018). They bind to free radicals, stimulate immune system or induce apoptosis, thus, prevent DNA damage, inhibit the activation and concentration of carcinogens, and restrict the development of neoplastic cells (Muszyńska et al. 2016). Three different polysaccharides, a water soluble (1–6) α‐D‐mannan and two types of β‐glucans, were isolated by Nyman et al. (2016) from *C. cibarius* mushroom. A unique polysaccharide (JP1) was purified by Chen et al. (2017) from the same species and found that it can facilitate the induction of peritoneal macrophages to release nitric oxide (NO), and increase the secretion of cytokines (IL‐6) in macrophage cell line (RAW264.7) of mouse. A new heteropolysaccharide (CC‐1) extracted from *C. cibarius* exhibited substantial in vitro antioxidant activity and immune cell proliferation effect (Zhao et al. 2018). The study revealed that CC‐1 has the potential to scavenge ABTS^+^ and DPPH free radicals at certain concentration levels.

### 4.2 Proteins and Amino Acids

Mushrooms are excellent source of proteins and play key role in both nutraceuticals and pharmaceuticals. The quantity of protein in mushrooms is nearly four times higher than that of tomatoes, six times that of oranges, and twelve times that of apples (Kakon, Choudhury, and Saha 2012; Chang 2003). One of the essential parts of Chantarelle is crude proteins. Ugbogu et al. (2020) have observed crude proteins content (13.71%–53.7%) in *Cantharellus* species. *C. cibarius* contain protein content (21.57 g/100 g) in dry mass of fruiting body (Ouzouni et al. 2009), and it was 9.3 mg g^−1^ DW of methanol extract (Kozarski et al. 2015).

The proteins like fungal immunomodulatory proteins (FIPs), antifungal and antimicrobial proteins (Lam and Ng 2001a, 2001b; Wong et al. 2010; Diling et al. 2017), ribonucleases (Kobayashi et al. 1992), ubiquitin‐like proteins (Lam, Ng, and Wang 2001; Zhou et al. 2017), ribosome‐inactivating proteins (RIPs) (Lam and Ng 2001a, 2001b; Wang and Ng 2001a, 2001b; Wong, Wang, and Ng 2008), lectins (Yagi et al. 1997; She, Ng, and Liu 1998; Marty‐Detraves et al. 2004), and laccases (Garzillo et al. 2001; Fukuda et al. 2001; Tinoco, Pickard, and Vazquez‐Duhalt 2001) are a few examples of mushroom proteins and these have interesting biological activities (Ng 2004; Xu et al. 2011). Several types of mushrooms contain lectins—the proteins that bind to carbohydrates. These mushroom protein “Lectins” have shown medicinal potentials including antitumor, antiviral, antifungal, antibacterial, immunomodulatory, and antiproliferative activity (Singh, Bhari, and Kaur 2010; Chatterjee, Halder, and Das 2021). The lectins found in chanterelle extracts typically agglutinate human type erythrocytes (Muszyńska et al. 2016). According to Lam, Ng, and Wang (2001), lectins and ubiquitin‐like proteins have antiproliferative effects on tumor cell lines and antimitogenic effects on spleen cells. They also have immunomodulatory and HIV‐1 RTase‐inhibitory effects (Wang et al. 1995; Wang and Ng 2001a, 2001b; Wu, Wang, and Ng 2011). A ubiquitin‐like peptide in *C*. *cibarius* possesses ribonucleolytic activity towards different polyhomoribonucleotides (Wang, Ngai, and Ng 2003).

The laccases are copper‐containing ligninolytic enzymes, and they have potential applications in biotechnology (Palmieri et al. 1993; Brenna and Bianchi 1994; Couto and Herrera 2006; Guest and Rashid 2016), like bio‐bleaching of paper pulp (Lu and Xia 2004; Camarero et al. 2004; Gutierrez et al. 2007; Camarero et al. 2007), bio‐catalysts in decolorization of synthetic recalcitrant dye (Hou et al. 2004; Camarero et al. 2005; Palmieri, Cennamo, and Sannia 2005; Michniewicz et al. 2008), detoxification or treatment of industrial effluents (Murugesan 2003; Dodor, Hwang, and Ekunwe 2004; Jaouani et al. 2005; Wu et al. 2008), and organic synthesis (Mikolasch et al. 2002; Karamyshev et al. 2003; Ncanana and Burton 2007). The mushrooms are rich source of laccase enzymes. The primary medicinal properties of laccases from basidiomycetes that have been documented so far include anticancer (Guest and Rashid 2016), antitumor, antibacterial, antioxidant, antidiabetic, and hypocholesterolemic activity (Jasim 2017). Antiproliferative activity of some mushroom proteins has been proved toward human hepatoma Hep G2 cells and T‐cell leukemia, and laccases are cytotoxic to breast cancer (MCF‐7) cells by targeting 17β‐estradiol (Xu et al. 2011; Guest and Rashid 2016). Ng and Wang (2004) isolated a homodimeric laccase from the sporocarp of *C. cibarius*.

Chanterelles contain high content of lysine and threonine amino acids (5.74 and 8.98 mg g^−1^ DW) (Beluhan and Ranogajec 2011). *Cantharellus* species contain glutamic acid (12.90 g), aspartic acid (7.74 g), leucine (7.56 g), and low tryptophan (0.92 g) per 100 g of protein (Ugbogu et al. 2020). Amino acid content was approximately 4.5% for *C. cibarius* (Panchak et al. 2020). Mushrooms are important source of a unique biomolecule, for example, ergothioneine, act as an essential antioxidant, improve health, and can be used as a food preservative, thus encouraging their use as functional foods. It is natural occurring amino acid and biologically derived from histidine (Richard‐Greenblatt et al. 2015; Rathore et al. 2019; Borodina et al. 2020). Ergothioneine was recorded 4.09 mg g^−1^ DW of *C. cibarius* (Martinez‐Medina et al. 2021). Fruiting bodies of several types of mushrooms are rich source of free amino acids. *C. cibarius* contains several types of L‐amino acids/mg mL^−1^ such as arginine (146.70), cystine (55.84), methionine (17.67), alanine (168.0), phenylalanine (72.10), lysine (65.80), valine (25.30), glycine (83.24), and leucine (65.74) (Salihovic et al. 2019). *Craterellus cibarius* contain alanine in higher amount, that is, 6.87 and 10.5 mg g^−1^ in fresh and dry, respectively. In *C. cibarius*, arginine present in higher amount, that is, 10.4 and 9.75 mg g^−1^ in fresh and dry weight, respectively (Salihovic et al. 2021).

### 4.3 Lipids

Mushrooms are rich in essential fatty acids (52%–87% USFA), especially linoleic acid, which the body unable to produce on its own, but required for health (Mokochinski et al. 2015; Chaturvedi et al. 2018). Polyunsaturated fatty acids (PUFA) are mostly found in edible mushrooms and enable them to significantly lower serum cholesterol (Chatterjee, Halder, and Das 2021). Majority of mushrooms contain PUFA, particularly linoleic acid, an essential omega‐6 fatty acid (Sande et al. 2019). Linoleic acid performs various physiological functions, including lowering blood pressure, triglyceride levels, cardiovascular disorders, and arthritis (Puttaraju et al. 2006; Ferreira, Barros, and Abreu 2009; Reis et al. 2012; Alves, Ferreira, Dias, et al. 2013; Alves, Ferreira, Froufe, et al. 2013). Fatty acids display remarkable antifungal and antimicrobial properties (Muszyńska et al. 2016). Edible mushrooms also contain important sterols (e.g., ergosterol) that may also be responsible for the antioxidant activity. According to research, sterols rich diet can help avoid cardiovascular problems (Barros et al. 2007; Kalac 2013). A fatty acid, that is, acetylenic acid isolated from *C. cibarius*, displays a significant transcriptional activity toward peroxisome proliferator–activated receptor γ (PPARγ), which controls inflammatory processes and cell growth and regulates metabolism of glucose and lipids (Muszyńska et al. 2016).

Fresh chanterelles contain ergosterol (24.7 mg), ergosta‐7‐enol (0.2 mg), and an equal amount (0.4 mg) of ergosta‐7,22‐dienol and ergosta‐5,7‐dienol (Muszyńska et al. 2016). The mushroom *C. cibarius* contain palmitic, palmitoleic, oleic, lauric, myristic, pentadecanoic, heptadecanoic, stearic, arachidonic, linoleic, behenic, cis‐8,11,14‐eicosatrienoic, cis‐11,14‐eicosadienoic, lignoceric, and tricosanoic acids. The major fatty acids were reported to be linoleic acid as 654.706 mg and oleic acid as of 148.168 mg (kg^−1^ DW); however, arachidonic acid was reported for the first time in *C. cibarius* (Ribeiro et al. 2009). High level of oleic acid (12.45%) has been recorded in *Cantharellus* species, followed by stearic acid (11.28%) and palmitic acid (8.05%) (Ugbogu et al. 2020).

The *C. cibarius* sporocarps contain a number of fatty acids, with 14,15‐dehydrocrepenyic acid being among the most prominent. It is found both as a free fatty acid and as a triglyceride. 14, 15‐dehydrocrepenyic acid is thought to be the precursor of cibaric acid (Pang and Sterner 1991). Linoleic acid (31.42%) reported to be a dominant component in hexane fraction of *C. cibarius* (Panchak et al. 2020). Ayaz et al. (2011) recorded 45.6% linoleic acid, 8.4% oleic acid, and the essential fatty acids in *Chantharellus cibarius* of Turkey. Kavishree et al. (2008) reported 20.8% linoleic acid and 25.9% oleic acid in *Cantharellus clavatus* from India. Ouzouni et al. (2009) reported that fruiting body of *C. cibarius* contains fat content (2.88 g) per 100 g dry fruiting body mass. Kolundzic et al. (2017) determined the various fatty acids, that is, MUFA (0.86%–45.43%) with oleic acid and cis‐vaccenic acid as principal fatty acids, followed by PUFA (0.53%–33.77%) having linoleic acid as the predominant fatty acid (0.50%–31.80%) in cyclohexane extract of *C. cibarius*. Along with fatty acid analysis, several types of ergosterol derivatives were identified by them in *C. cibarius*, that is, ergosta‐5,7,9 (Kozarski et al. 2015); 5,6‐dihydroergosterol; 22‐tetraen‐3b‐ol; ergosta‐4,6,8; ergosta‐5,7‐dien3‐ol; ergosterol; 22‐tetraen‐3‐one; and ergosterol acetate, euphorbol, and lanosterol as well. Chanterelle contains more saturated fatty acid (SFA) that accounts for 926.953 mg/kg dry matter followed by PUFA (655.176) and MUFA (148.493) (Ribeiro et al. 2009). Monounsaturated fatty acids (MUFA) and polyunsaturated fatty acids (PUFA) are more potent fungicides in comparison with saturated fatty acids (SFA). Additionally, its apricot‐like and cooked carrot‐like odor is due to aromatic C_8_ volatile compounds, that is, 3‐octanone, (E)‐2‐ octenol, (E)‐2‐octenal, and octanal (Aisala et al. 2019), and 1‐octen‐3‐ol that is synthesized during free linoleic acid oxidation reaction catalyzed by oxidoreductases. This reaction is intensified, particularly during drying process (Kalac 2009).

### 4.4 Vitamins and Minerals

Edible mushrooms are rich wellspring of nutritionally significant vitamins (group B, C, D, and E) (Vetter 1994; Mattila, Suonpää, and Piironen 2000; Mattila et al. 2001; Heleno et al. 2010; Kalaras, Beelman, and Elias 2012). The B‐vitamins that are most frequently recorded in edible mushrooms include thiamine, riboflavin, pyridoxine, nicotinic acid, pantothenic acid, folic acid, cobalamin, and nicotinamide (Assemie and Abaya 2022). In today's scenario, oxidative stress contributes to the progression of numerous chronic conditions, such as diabetes and cancer (Ceriello and Motz 2004), as well as other cardiovascular diseases (Heitzer et al. 2001). Recent research suggested that antioxidants can control autoxidation by inhibiting or preventing the free radical proliferation and thus subsequently reduce oxidative stress, boost immune function, and lengthen healthy lifespan (Tan et al. 2018). Vitamins C and E, tocopherols, and carotenoids react with free radicals, especially peroxyl radicals and singlet molecular oxygen, which is the basis of their antioxidant activity (Sies, Stahl, and Sundquist 1992). There are few overlooked antioxidant vitamins that include vitamin D, vitamin K, riboflavin, pyridoxine, and niacin which function as coenzymes to combat free radicals, and their deficiencies hasten the development of oxidative stress (Sinbad et al. 2019). The substances that are responsible for antioxidant activity also have a variety of biological effects, including the protection from malignancy, heart diseases, and various degenerative disorders (Chatterjee, Halder, and Das 2021). Vitamin E has potential to prevent DNA damage and reduce the risk of cardiovascular diseases, which is due to its antioxidant, anti‐inflammatory, and anticancer activities (Jiang 2014). Number of researchers are aware with salient functions of various vitamins like, Phytonadione (vitamin K) as neuroprotective (Li et al. 2003), riboflavin and calcitriol control lipid peroxidation and protein carbonylation (Wang et al. 2011; AlJohri, AlOkail, and Haq 2018), and vitamin B12 increase viability of retinal ganglion cells (Chan et al. 2018).

Chanterelle mushrooms have a wealth of vitamins A and E. In nature, vitamin E occurs in eight different forms: α‐, β‐, γ‐, and δ‐tocotrienol and α‐, β‐, γ‐, and δ‐tocopherol (Barros et al. 2008; Jiang 2014). Ergocalciferol, tocopherols, and carotenoids have also been reported in the chanterelle, and the tocopherol content was reported to be approximately 0.82% (Panchak et al. 2020). Fresh chanterelle is also a good source of vitamin D2 (14.2 μg/100 g FW), while dried sporocarps hold 0.12–6.3 μg g^−1^ of ergocalciferol after 2–6 years of storage (Muszyńska et al. 2016). *C. cibarius* contain high content of ergocalciferol as equivalent to those in fish. Ergocalciferol concentrations in different dried fruiting bodies varied from 0.12 to 6.30 μg g^−1^ (Rangel‐Castro, Staffas, and Danell 2002), and the folic acid content of *C. cibarius* was reported 5.07 g/100 g in *C. cibarius* by Egwim, Elem, and Egwuche (2011). Vitamin A level recorded the highest (16.65 IU/100 g) in quantity as compared to other vitamins (B1, B2, B3, B6, B12, and C) in *Cantharellus* species (Ugbogu et al. 2020). Fresh mass of *C. cibarius* contains high content of vitamin C (1.95 mg g^−1^) and low (0.52 mg g^−1^) in dry mass (Salihovic et al. 2021).

Mushrooms collected from natural habitats are the common constituents of nutritionally valuable minerals (Falandysz and Borovička 2013), like, Se, Cu, K, P, Mg, Mn, Na, Fe, Ca (Mattila et al. 2001), and zinc that are required for proper regulation of metabolic pathways (Das et al. 2021). Minerals from the growth media are accumulated in the sporocarps of mushrooms (Rajarathnam, Shashirekha, and Bano 1998; Kalac 2010). Attempts are being made in biofortification of cultivated mushrooms with biologically active and medicinally important minerals, like Se, Li, and Fe (da Silva et al. 2010; Hong et al. 2011; De Assuncao et al. 2012; Kaur, Kalia, and Sodhi 2017; Budzyńska et al. 2022). Moreover, selenium acts as a strong antioxidant mineral (Jayachandran et al. 2018).

Falandysz and Drewnowska (2015) provided information of elements found in *C. cibarius* from Poland. Similar investigation was performed by Drewnowska and Falandysz (2015) with the same species. Fruiting bodies of chanterelle investigated from all places were comparatively rich in essential elements, viz., K, P, Cu, Mg, Zn, Mn, Ca, Na, Co, and Fe. This mushroom, that is, *C. cibarius*, efficiently accumulates K, P, Cu, Na, Rb, Cd, Ag, and Zn from the soil. A high content of calcium was recorded in *C. cibarius* followed by K and P (Wang et al. 2014; Kolundzic et al. 2017). Ugbogu et al. (2020) reported highest level of Ca^2+^ions followed by K^+^ and Fe^2+^ ions in *Cantharellus* species.

Additionally, mushrooms are regarded as promising bioremediation agents for soils contaminated with heavy metals, with many edible species capable of efficiently accumulating these metals in their tissues (Drewnowska et al. 2017). The presence of chemical elements in food, both essential and harmful to humans, such as As, Cd, Hg, and Pb, raises significant concerns among consumers (Drewnowska and Falandysz 2015). *C. cibarius* is known to accumulate heavy metals like Cu, Cd, Hg, and Pb (Falandysz et al. 2012; Drewnowska et al. 2017; Balta et al. 2018; Uzun et al. 2023). Therefore, techniques for reducing heavy metal concentrations prior to consumption are essential. According to research, blanching fresh chanterelles can decrease cadmium (Cd) levels by approximately 11% ± 7% to 36% ± 7%, while blanching frozen mushrooms can reduce Cd content by about 40 ± 6%. Both blanching and pickling are shown to significantly lower Cd levels in *C. cibarius* (Drewnowska et al. 2017). When sliced fruiting bodies of *Imleria badia* were boiled under reflux for 15–60 min, cadmium (Cd) leaching was more effective in frozen mushrooms (58%) compared to fresh ones (36%), with boiling duration having no significant effect. For *C. cibarius*, simply washing the halved fruiting bodies under running tap water for about 45 s prior to cooking increased the amount of liquid released and enhanced the removal of radionuclides, specifically ^137^Cs and ^134^Cs, by approximately 30% during cooking (with vegetable oil in a pan) (Steinhauser and Steinhauser 2016).

## 5 Nutraceuticals and Medicinal Profile

“Nutraceuticals” term was first time coined in 1989 by Stephen De Felice (Kalra 2003). According to Daliu, Santini, and Novellino (2018), “A product could be considered as nutraceutical, if it has a positive effect on health, confirmed by clinical testing.” Nutraceuticals are dietary supplements having noteworthy health benefits and also prevent the onset of several maladies (Chatterjee, Halder, and Das 2021) by strengthening human performance (Chang and Buswell 1996; Prasad et al. 2015; Ma et al. 2018).

Medicinal mushrooms are defined as those that produce secondary metabolites and have a variety of biological purposes. Approximately 270 different kinds of mushrooms have therapeutic potential (Shamtsyan 2010), and only a small proportion of mushrooms are considered as nutraceutical mushrooms (Wu et al. 2019). Mushrooms have been used as food and even as medicine for thousands of years (Beelman, Kalaras, and Richie 2019), particularly in Asian countries (Japan, China, and Korea), as well as in some regions of Africa (Ozturk et al. 2015). These days many of them are being used against immune disorders and cancer risk as a supplementary medicine (Novak and Vetvicka 2008; Ooi 2008; Wasser 2011; Ren et al. 2012). In addition to their nutritional, delicacy, and functional qualities, mushrooms are considered as nutraceutical foods having medicinal and organoleptic properties (Ergönül et al. 2013; Phat, Moon, and Lee 2016; Rahi and Malik 2016). Based on bioactive properties of mushrooms, they have become popularized as a functional food, pharmaceuticals, and nutraceuticals (Jones and Janardhanan 2000; Lakhanpal and Rana 2005).

Mushrooms contain several types of nutraceuticals, such as lectins, lentinan, β‐glucan, proteoglycan, ganoderic acid, phenolics, triterpenoids, flavonoids, hispolon, calcaelin, laccase, ergosterol, nucleosides, and nucleotides (Patel and Goyal 2012; Ina, Kataoka, and Ando 2013; El Enshasy and Hatti‐Kaul 2013; Papoutsis et al. 2020). They also contain protocatechuic, p‐hydroxybenzoic, vanillic, syringic, gentisic, cinnamic, veratric, salicylic, p‐coumaric, gallic (Alves, Ferreira, Dias, et al. 2013; Alves, Ferreira, Froufe, et al. 2013), caffeic, and ferulic acids (Nowacka‐Jechalke, Olech, and Nowak 2018). In general, on the basis of chemical compositions and interactions with biochemical functions, mushroom nutraceuticals exhibit a variety of biological functions, which include anticarcinogenic, antitumor, anti‐inflammatory, antimycotic, anti‐obesity, antidiabetic, antibacterial, antiviral, antihypercholesterolemic, and antimutagenic (Borchers et al. 2008; Zhang et al. 2009; Valverde, Hernández‐Pérez, and Paredes‐López 2015; Reis et al. 2017).

**FIGURE 2:**
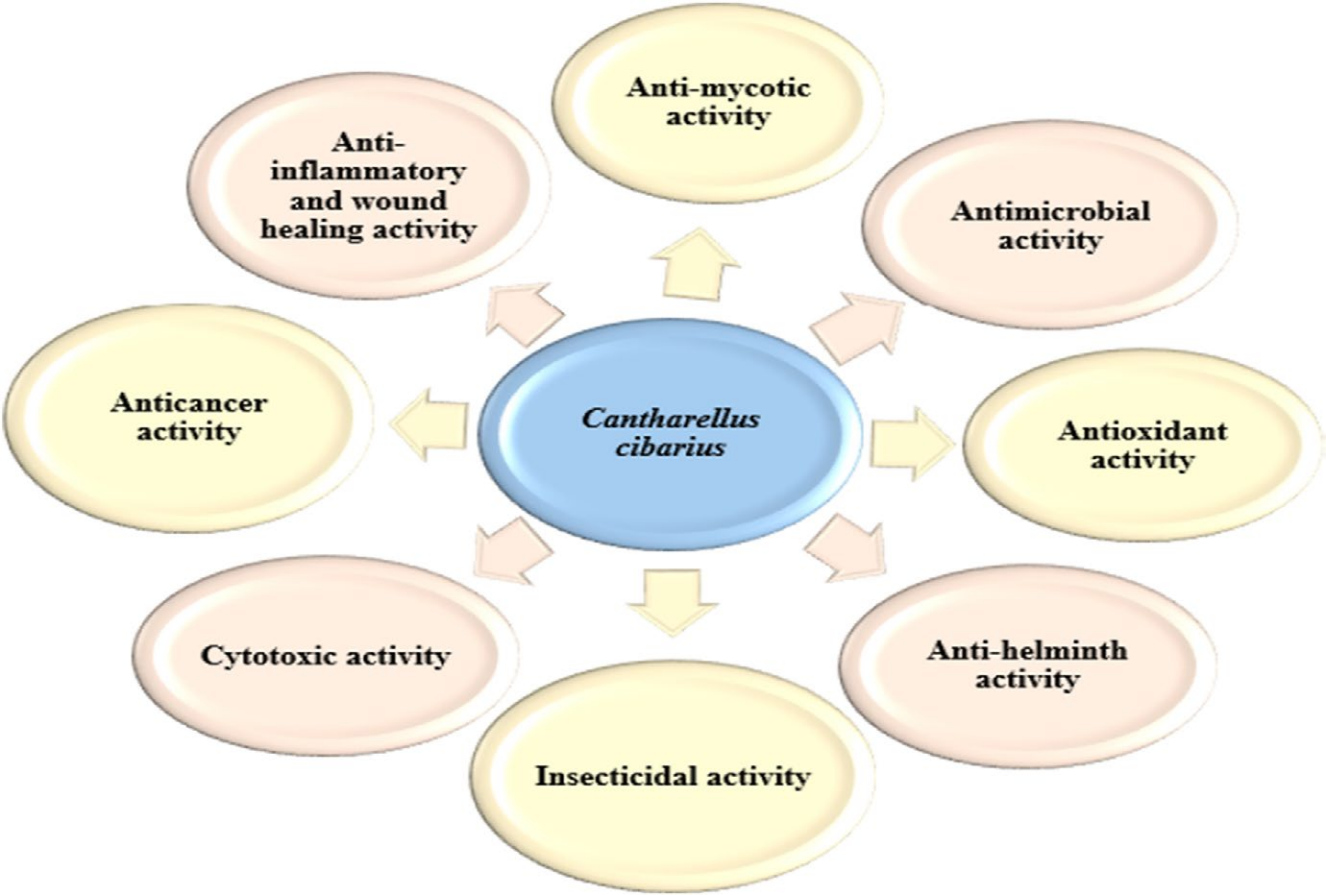
Biological activities of *C. cibarius*.

*C. cibarius* is used in the treatment of boils and various types of abscesses and also acts as antihelmintic (Panchak et al. 2020). Moreover, decoction prepared from chantarelle mushrooms is employed as a food adjuvant in making functional frankfurters with high antimicrobial and antioxidant qualities as well as low sensorial alterations. Chantarelles are enriched with antioxidants and thus could be utilized in food products like cooked pork sausages to prevent chemical degradation and to enhance their shelf life (Novakovic et al. 2019; Novaković et al. 2020). *C. cibarius* is rich in minerals, carbohydrates, and free amino acids that are involved in maintaining immune system functional. Several biological properties like antioxidant, anti‐inflammatory, antimicrobial, antihypoxic, antihyperglycemic, wound‐healing, cytotoxicity, and iron‐chelating activity are shown by *C. cibarius* (Figure 2) (Vlasenko et al. 2019). The review has covered the most common medicinal properties of *C. cibarius*, which include antimicrobial, antimycotic, antihelminth, antihypoxic, cytotoxicity, antihyperglycemic, anticancerous, antioxidant, anti‐inflammatory, iron‐chelation, and wound‐healing properties.

### 5.1 Bioactive Compounds and Pharmacological Properties

Bioactive substances are the molecules with therapeutic potential that can improve energy intake, lower down pro‐inflammatory states, reduce oxidative stresses, and overcome metabolic disfunctions (Siriwardhana et al. 2013), and thus ultimately promote better health. Bioactive compounds can influence metabolic reactions and exhibit antioxidant activity, inhibition of receptors, suppression or induction of enzymes, and gene expression (Carbonell‐Capella et al. 2014). There has been a growing interest in biologically active substances of mushrooms that have therapeutic or health benefits for mankind in curing and preventing various diseases (Rathee et al. 2012). The bioactives of mushrooms are polysaccharides (e.g., β‐glucans) and cell wall proteins, or phenolics, terpenes, and steroids as secondary metabolites (Sánchez 2017).

Medicinal mushrooms contain some unique polysaccharides that prevent the cancer like life‐threatening disease and have immunomodulatory property which strengthen the immune system (Ozturk et al. 2015). The polysaccharides have potential role in modern medicine, where β‐glucan regarded as a flexible metabolite confer a broad spectrum of biological functions (Patel and Goyal 2012; Chang and Wasser 2012; El Khoury et al. 2012; Finimundy et al. 2013). Polysaccharides of *C. cibarius* have numerous bioactivities such as antitumor, anticancer, antioxidant, immune‐modulatory, immune‐stimulatory, neuroprotective, antiproliferative, prebiotic, and chemopreventive. Moreover, its crude extracts also have antioxidant, antihyperglycemic, antihyperlipidemic, antihypertensive, antiinflammatory, antiangiogenic, antigenotoxic, antihypoxic, cardioprotective, antimicrobial, antiviral, LOX inhibition, cytotoxic, wound‐repairing, and age delaying properties (Muszyńska et al. 2016; Nasiry, Khalatbary, and Ebrahimzadeh 2017; Nowacka‐Jechalke, Olech, and Nowak 2018; Turfan et al. 2019; Badalyan and Rapior 2020; Thu et al. 2020; Uthan et al. 2021; Marathe et al. 2021).

Several studies have documented mushrooms as natural affluent sources of phenolics and flavonoids (Manzi et al. 2004; Valentao et al. 2005; Barros et al. 2008; Nowacka et al. 2014; Woldegiorgis et al. 2014; Heleno, Martins, et al. 2015; Sarikurcku et al. 2015), and these are important groups of biologically active compounds in the mushrooms (Oskoueian et al. 2011). Phenolics mainly including the phenolic acids, lignans, hydroxycinnamic acids, tannins, hydroxybenzoic acids, stilbenes, flavonoids, and oxidized polyphenols are found in mushrooms (D'Archivio et al. 2010; Thu et al. 2020). The phenolic compounds display antioxidant, antiviral, antibacterial activities, reduce inflammations, and protect from carcer (Silva et al. 2004; Soobrattee et al. 2005; Reis et al. 2011; Wang et al. 2014; Heleno, Ferreira, et al. 2015; Heleno, Barros, et al. 2015). A few studies (Reis et al. 2012; Smolskaitė, Venskutonis, and Talou 2015) have correlated the polyphenols with antioxidant activity. In mushroom extracts, phenolic compounds function as antioxidant by acting as peroxidase decomposers, metal inactivators, oxygen scavengers, or free radical inhibitors (Dziezak 1986).

The chanterelle mushrooms contain various kinds of phenolic compounds and organic acids (Valentao et al. 2005). *C. cibarius* contain major groups of primary as well as secondary bioactive metabolites, for instance, flavonoids, phenolic acids, sterols, fatty acids, organic acids, indole groups, carbohydrates, vitamins (tocopherols), amino acids, enzymes, bioelements, carotenoids, and 5ˊ‐nucleotides (Nyman et al. 2016; Muszyńska et al. 2016; Thu et al. 2020; Panchak et al. 2020). These bioactive components might be utilized in pharmaceuticals or nutritional adjuncts (Muszyńska et al. 2016).

Flavonoids are natural compounds with a polyphenolic structure. They play key role in cellular enzymatic functions because of their antioxidant, antimutagenic, anticarcinogenic, anti‐inflammatory (Panche, Diwan, and Chandra 2016), and cardioprotective function that generally associated with their antioxidant properties (Aaby, Hvattum, and Skrede 2004). Flavonoids are considered highly efficient free radical scavengers among several oxidizers, act against ROS and many other free radicals that are probably responsible for DNA damage and tumorigenesis (Le Marchand 2002). In addition to phenolics, mushrooms including chanterelle have been reported to contain flavonoids that scavenge free radical and ultimately block the radical reactions that occur at the time of triglyceride oxidation in the food system (Barros et al. 2008). The most susceptible cellular components that can be damaged by free radicals are lipids (by peroxidation process), proteins (by denaturation process), and nucleic acids (disturbing normal cell cycle). Protein denaturation is a major reason of inflammation and leads to the onset of rheumatoid arthritis. *C. cibarius* extracts show erythrocyte membrane stabilization effect and antiproteinase activity. The release of lysosomal constituents causes inflammation and damage which could be reduced by stabilizing the lysosomal membrane (Siju et al. 2015).

#### 5.1.1 Antimicrobial and Antimycotic Activity

Mushrooms might be an alternative option for novel antimicrobial compounds. They provide several types of primary (peptides, proteins, and oxalic acid) as well as secondary metabolites including steroids, terpenes, and quinolones (Valverde, Hernández‐Pérez, and Paredes‐López 2015). *C. cibarius* exhibits antibacterial and antifungal attributes (Dulger, Gonuz, and Gucin 2004; Barros et al. 2008; Santoyo et al. 2009; Ramesh and Pattar 2010; Aina et al. 2012; Alves, Ferreira, Dias, et al. 2013; Alves, Ferreira, Froufe, et al. 2013) and trypanocidal attributes (Ustun, Kaiser, and Tasdemir 2011; Abedo et al. 2015). The acetone and methanol extract of *C. cibarius* also had antimicrobial activity (Aina et al. 2012; Kosanic, Rankovic, and Dasic 2013) and also reported for its liquid culture (Popova 2015). High cidal activity of phenolic acids has been reported against the majority of bacterial groups. Phenolic acids (2,4‐dihydroxybenzoic, vanillic, syringic acids) inhibited more methicillin‐resistant *Staphylococcus aureus* (MRSA) at MICs (0.5 mg/mL) than methicillin sensible *Staphylococcus aureus* (Alves, Ferreira, Dias, et al. 2013; Alves, Ferreira, Froufe, et al. 2013).

Mushroom could be used in prevention and treatment of *Helicobacter pylori* infection because of their valuable compounds (polyphenols and polysaccharides). All different extracts of *C. cibarius* performed dynamically at varying degree of MICs (62.5–250 μg/mL). Minimum inhibitory concentrations (4–32 μg/mL) were recorded for methanolic extract against *H. pylori* strains (Kolundzic et al. 2017). The decoction of *C. cibarius* displayed bactericidal activity at maximum concentration (20 mg/mL) toward *Yersinia enterocolitica* and *Listeria monocytogenes* . The fungistatic and fungicidal activity of decoction was significantly better against *Candida albicans* at MIC/MFC (10 mg/mL) (Novakovic et al. 2019).

#### 5.1.2 Anticancerous and Cytotoxic Activity

**FIGURE 3:**
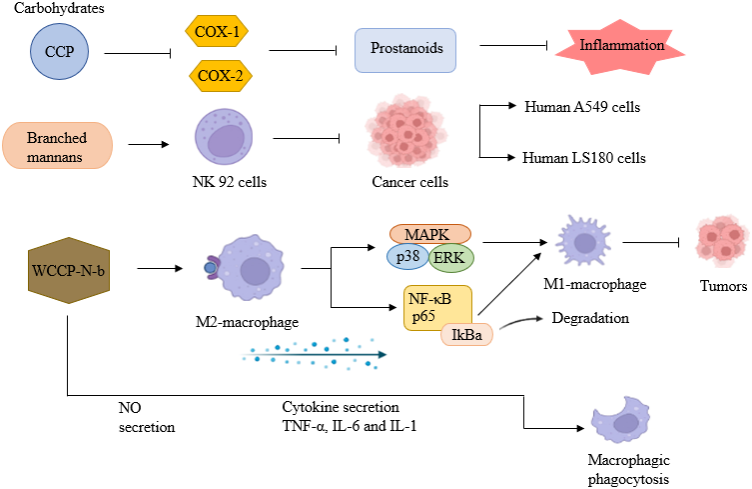
Different activities of carbohydrates isolated from *C. cibarius* (CCP, Cantharellus crude polysaccharide; COX, cyclooxygenase; NK, natural killer cell; WCCp‐N‐b, a polysaccharide; Human A549 and LS180 cancer cell line).

About 660 species of higher basidiomycetes possess antitumor activity (Zaidman et al. 2005). As well‐known that edible mushrooms have numerous bioactive compounds and polysaccharides are one of the main constituents (Chen et al. 2018; Sharma, Singh, and Singh 2018). These polysaccharides have the ability of generating innate immune responses by boosting NO secretion and expression of pro‐inflammatory interleukins (IL‐1, IL‐6, IL‐10, IL‐12, and TNF‐α) in macrophages (Wang and Mazza 2002; Doyle and O'Neill 2006; Lai, Yang, and Lin 2015; Han et al. 2015; Zhang et al. 2022; Ye et al. 2020). A polysaccharide (CCP) from *Craterellus cornucopioides* is reported to activate the TLR4–NFκB pathway at concentration (40 μg/mL), which led to the increase in phagocytic function, expression of receptor (TLR4), production of cytokines, and protein kinases (Guo et al. 2020). Homopolymer and extremely complex heteropolymer glycans show antitumor activity (Chatterjee, Halder, and Das 2021). The phenomenal activities of polysaccharides obtained from *C. cibarius* are shown in Figure 3.

The enzyme cyclooxygenase (COX) is responsible for producing the prostanoids, that is, prostaglandins, prostacyclins, and thromboxanes—that cause inflammation (Ricciotti and FitzGerald 2011). Anti‐inflammatory therapeutics might be responsible for the deactivation of two (COX‐1 and COX‐2) isoforms of COX enzymes. Interesting chemopreventive potential is present in *C. cibarius*, particularly in the treatment of colon cancer. This chanterelle species contains a unique monosaccharide with having the potential to inhibit the activation of COX‐1 and COX‐2 isoforms (Nowacka‐Jechalke, Olech, and Nowak 2018), but does not show any cytotoxicity toward normal human colon (CCD 841 CoTr) cells across the entire applied concentrations. A methanolic extract of *C. cibarius* is selectively cytotoxic to human cervix adenocarcinoma cells (HeLa line), K562 cell line, and MDA‐MB‐453 cell line as compared to normal lung cells (Kozarski et al. 2015). But it was observed that *C. cibarius* extracts (cyclohexane and dichloromethane) equally affect HeLa, N87 cells, and healthy MRC‐5 cells (Kolundzic et al. 2017). Cytotoxic effects of the extracts were also assessed toward primary mammalian L6 cells (Ustun, Kaiser, and Tasdemir 2011). Methanolic extracts of *C. cibarius* also exhibit high cytotoxic activity and induce apoptotic necrosis in A549 cells. Piceatannol an analog of resveratrol is identified from this mushroom as an active ingredient which is known for antiproliferative activity (Vasdekis et al. 2018).

Polysaccharides from medicinal mushrooms can activate macrophages, cytotoxic lymphocytes (NK cells), leukocytes (neutrophils), and induce gene expression of cytokines and interleukins (Zhao et al. 2020). Mushrooms contain a water‐soluble polysaccharide (β‐glucan) that triggers attack on tumor cells by T cells, NK cells, macrophages, and cytokines (Vetvicka et al. 2008). Branched mannans could be a new option in fighting colon cancer. Branched mannan obtained from CC2a fraction of *C. cibarius* enhances survival, proliferation, and anticancer property of human natural killer cells (NK92) against the human A549 and LS180 cancer cell line (Lemieszek et al. 2019; Lemieszek, Nunes, and Rzeski 2019). NF‐κB activity stimulates proliferation of tumor cells and suppression of apoptosis, initiates angiogenesis, and activates epithelial–mesenchymal transition that promotes distant metastasis (Xia, Shen, and Verma 2014). Sporocarps of *C. cibarius* contain active metabolites such as ergosterol, ergosterol peroxide, cerevisterol, β‐sitosterol, 7‐dehydrostigmasterol, tuberoside, glucoside, and cerebroside, which can inhibit NF‐κB activation by preventing it from moving from the cytoplasm to the nucleus. (Kim, Tay, and de Blanco 2008).

Macrophages are component of the innate immune system, carry out immunological surveillance and tumor defense. Tumor‐associated macrophages (TAMs) found in the tumor microenvironment and are segregated into two groups: M1 and M2 macrophages (Nielsen and Schmid 2017). Macrophages are one of the main target cells of some antitumor and immunomodulatory drugs. Tumor cell invasion, proliferation, and metastasis are associated with TAMs with M2‐like phenotype. As a result, they might be a suitable target for an efficient cancer immunotherapy. A new polysaccharide WCCP‐N‐b (linear α‐1,6‐galactan) has been purified from *C. cibarius* by Yang et al. (2019) and has the ability to switch M2‐like macrophages (tumor‐promoting) to tumor‐inhibiting M1‐like phenotype (Meng et al. 2019). This linear galactan has the ability to greatly boost phagocytosis by macrophages, NO secretion, and production of cytokines (TNF‐ α, IL‐6, and IL‐1) and also activates different signaling pathways (NF‐κB and MAPKs).

Numerous edible mushrooms have phytochemicals that are known to have antitumor properties. The viability of cancer cells in four cancer cell lines (U87 glioblastoma, A172 glioblastoma, PAN‐C1 pancreatic, and CH157‐MN meningioma) and one NIH3T3 fibroblast were dramatically reduced by high doses (1000 and 2000 μg/mL) of *C. cibarius* extracts (Chin et al. 2018). Such reduction in cancer cells could be credited to the occurrence of β‐glucans and phenolic compounds (Kolundzic et al. 2017), which exhibited antioxidative (Wang et al. 2014), as well as antiangiogenic activities (Kao et al. 2013). Han et al. (2013) isolated a protein‐bound polysaccharide fraction (JBP‐1), that is, water‐soluble and protein‐bound glucan from the sporocarps of *C. cibarius*. JBP‐1 possesses immunomodulatory potential when lymphocytes were selected to evaluate the immunological activity. This polysaccharide functions as an immunocompetent adjuvant and could be further used for clinical applications, such as treatment for cancers.

Various chronic degenerative diseases, including cancer, rheumatoid arthritis, diabetes, cardiovascular disease, and chronic inflammation are caused by genotoxins (Izquierdo‐Vega et al. 2017). Aqueous extract of *C. cibarius* showed antigenotoxic potential against damage induced by the alkylating carcinogenic agent (methyl methanesulfonate) in human mononuclear cells (hMNCs) (Méndez‐Espinoza et al. 2013).

**FIGURE 4:**
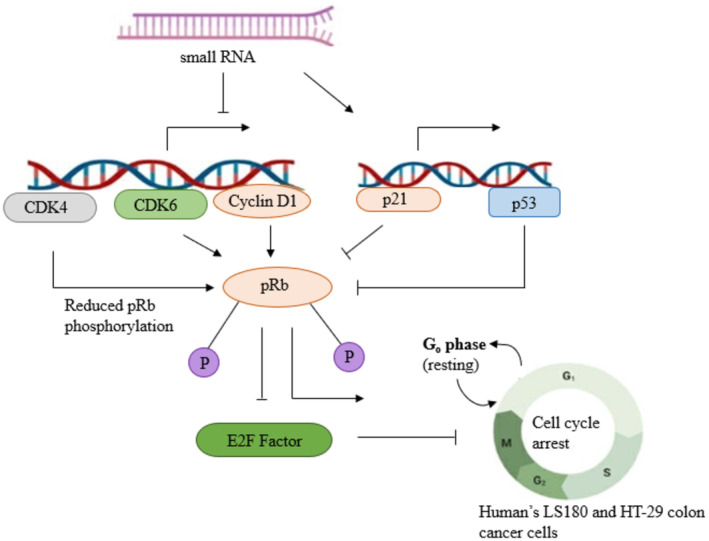
Function of sRNA isolated from *C. cibarius* in cell cycle arrest in cancer tissues (CDK: Cyclin‐dependent kinase; pRb: Phosphorylated retinoblastoma protein).

Small RNAs from *C. cibarius* (Figure 4) have proved strong antiproliferative activity against human's LS180 and HT‐29 colon cancer cell lines (Lemieszek et al. 2019), while nontoxic effect was observed for CCD841 CoTr human colon epithelial cells. This happens due to the elevated p53 expression and p21‐mediated p53‐dependent cell cycle arrest. Ethanolic extract (80%) of C. *cibarius* exhibits antiproliferative activity when applied at 0.1 mg mL^−1^, and aqueous extract is weak immuno‐stimulatory at 1 mg mL^−1^ (Deo et al. 2019).

#### 5.1.3 Insecticidal Activity

Insecticidal activity of isolated glycerol 1, 2, and 1, 3‐dilinoleates and glycerol tridehydrocrepenynate from *C. cibarius* was examined against *Musca domestica* and *Blatta orientalis* by Daniewski et al. (2012). Ethanolic extract fraction showed that insecticidal activity occurred mostly in the nonpolar compounds found in the ethyl acetate layer. Insecticidal activity of the further isolated ethyl acetate fraction was reported lowered than that of the crude extracts. This finding may indicate a synergistic interaction of the constituents of the chanterelle (*C. cibarius*). Cieniecka‐Rosłonkiewicz et al. (2007) revealed high insecticidal activities of protic ionic liquid extract of *C. cibarius* against both *Musca domestica* and *Blatta orientalis*.

#### 5.1.4 AntiHelminth Activity

Extract of *C. cibarius* is also used as an anthelmintic agent (Panchak et al. 2020). Fascioliasis originally affects livestock, but it is now known to affect humans. Ethanolic extract of *C. cibarius* has ovicidal and miracicidal activities against *Fasciola* spp. (Nwofor et al. 2018). Opisthorchiasis is a dangerous disease caused by *Opisthorchis felineus* (trematode) of the family Opisthorchiidae, which is common in Western Europe and Russia. There are a lot of complications associated with this disease and relatively have few effective treatments. Methanolic extract of *C. cibarius* dramatically reduced the population of *O. felineus* in mice bile ducts with increasing concentrations (10–1000 μg/mL) (Tsyganov et al. 2018), if applied on parasite larvae excyst.

#### 5.1.5 Antioxidant Activity

Antioxidants have great importance in terms of combating oxidative stress that may cause numerous degenerative diseases (Helen et al. 2000). Phenolics are the principal compounds in mushrooms responsible for their antioxidant activity (Elmastas et al. 2007). Valentao et al. (2005) listed various phenolics and organic acids from *C. cibarius*. Antioxidant activity of these substances might serve as a defense against a variety of diseases (Mier et al. 1996). The potencies of phenolics to scavenge unstable molecules (free radicals), chelate metal ions, and inhibit lipoxygenase (LOX) enzyme may be responsible for their bioactivities (Decker 1997; Mallavadhani et al. 2006). Witkowska, Zujko, and Mirończuk‐Chodakowska (2011) explored most popular wild edible mushrooms including *C. cibarius* as rich sources of antioxidants such as total polyphenol contents. Polyphenol content in *C. cibarius* was found 270 mg/100 g in dry mass and 22 mg/100 g in fresh mass. DPPH radical scavenging ability was reported in EC_50_ values as 13.86 mg extract/mL, and chelating ability of ferrous ions was valued EC_50_ 8.02 mg extract/mL. In the methanolic extract of *C. cibarius*, Kozarski et al. (2015) reported phenols (49.8 mg g^−1^) as the major antioxidant component followed by flavonoids (42.9 mg g^−1^). Tekeli, Dogan, and Uslu (2008) determined antioxidant activity of *C. cibarius* extract, which showed a higher potency than butylated hydroxytoluene (BHT) and butylated hydroxyanisole (BHA) in scavenging of DPPH free radical.

The carotenoid pigments have antioxidant properties which have been an accountable factor for positive effects on human health (Rao and Rao 2007). Particularly β‐carotene has shown promising results in laboratory assays and observed inverse association with cancer threat in epidemiologic studies (Barros et al. 2008). A peculiar biomolecule, that is, ergothioneine, is present in *C. cibarius* and has been regarded as a crucial biological antioxidant because of the way it cooperates with other antioxidants to prevent oxidative stress in mitochondria. Additionally, this amino acid enhances deactivation of singlet oxygen (Akanmu et al. 1991) and acts as a protective shield against oxidative damage of water‐soluble proteins (Paul and Snyder 2010).

The mushroom *C. cibarius* have the power of inhibition of lipid peroxidation reaction at 49.74 nM, which emphasizes its antioxidant potential (Egwim, Elem, and Egwuche 2011). Many studies have reported that the presence of flavonoids might be responsible for their antioxidant properties (Harborne and Williams 2000) and may be because of its antilipid peroxidation activity in some of the studied mushrooms. Based on linoleic acid autoxidation, maximum inhibition (74%) was reported for *C. cibarius* by Palacios et al. (2011). Higher flavonoids (40.01 mg QE g^−1^ of extract) and phenol content (40.97 mg GAE g^−1^ of extract) in n‐butanol fraction were observed in *C. cibarius* by Ebrahimzadeh, Safdari, and Khalili (2015). They also observed highest nitric oxide (NO) scavenging activity; higher DPPH scavenging activity (33.43%) in ethyl acetate fraction; high reducing potential in aqueous fraction; and highest Fe^2+^ chelating activity (86.13) in the chloroform fraction. In addition, *C. cibarius* fractions (CC2a, CC3) have shown antioxidant activity (Lemieszek et al. 2018).

#### 5.1.6 Anti‐Inflammatory and Wound‐Healing Activity

The application of *C. cibarius* in wound treatment might be owing to its potent anti‐inflammatory and wound‐healing actions (Nasiry, Khalatbary, and Ebrahimzadeh 2017). Numerous neurodegenerative pathways have been intimately correlated with inflammatory mechanisms (Chen, Zhang, and Huang 2016). The anti‐inflammatory phytochemicals of *C. cibarius* may be utilized effectively in neuroprotection. Neuroprotective potential of polysaccharide fractions of *C. cibarius* was examined under different neurodegeneration models, including excitotoxicity, trophic, and oxidative stresses. The research revealed positive effects of *C. cibarius* fractions on neuron viability and neurite development under both stress and normal conditions. These both types of fractions (CC2a and CC3) efficiently counteract the detrimental effects induced by glutamatergic system activators (Lemieszek et al. 2018). Methanolic extract of *C. cibarius* exhibits anti‐inflammatory activity revealed by using RAW 264.7 macrophages (Moro et al. 2012). In response to LPS stimulation, the extracts induced suppression of nitric oxide (NO) and upregulate mRNA expression of iNOS and cytokines (IL‐1b and IL6). Palacios et al. (2011) detected pyrogallol in the extracts of *Agaricus bisporus*, *C. cibarus*, *C. cornucopioides*, and *Lactarius deliciosus* and suggested that it might have some anti‐inflammatory activities.

The peroxisome proliferator–activated receptors (PPARs) are members of nuclear receptor proteins that play vital role in the regulation of energy metabolism (Christofides et al. 2021), proliferation, differentiation, inflammation (Clarke et al. 1999; Clark 2002; Peters, Shah, and Gonzalez 2012), and tumorigenesis (Belfiore, Genua, and Malaguarnera 2009). A new acetylenic acid analogue (10E,14Z)‐9‐oxooctadeca‐10,14‐dien‐12‐ynoic acid was isolated from *C. cibarius*, which can selectively activate PPAR‐γ (Hong et al. 2012). These nuclear receptors play crucial role in development of adipocytes, regulation of inflammatory processes, in lipid and carbohydrate metabolism (Muszyńska, Sułkowska‐Ziaja, and Ekiert 2011).

## 6 Culinary Delicacy

Mushrooms have been a key part of the human diet since the prehistoric era. Mushrooms were even regarded as “Food of the Gods” by the Romans (Chatterjee, Halder, and Das 2021). It was believed by the Greek, that mushrooms provide strength during the battle and were considered as a health food or “Elixir of Life,” according to Chinese (Valverde, Hernández‐Pérez, and Paredes‐López 2015). Chanterelles commonly live in symbiotic association with trees of pine, spruce, hornbeam, and oak. They generally grow from the month of June to October. They are well regarded as a preferred delicacy in America, Africa, and Asia and in several European countries (Bulam, Ustun, and Peksen 2021). Wild *C. cibarius* mushroom has been found to have medicinal and health benefits, which may be another factor in its use as traditional delicacy (Kozarski et al. 2015). Through conventional culinary techniques (frying, cooking, and marination), the juvenile sporocarps can be consumed and preserved by drying, freezing, and other methods (Muszyńska et al. 2016; Sumic et al. 2016; Thu et al. 2020). Chantarelles are being served with chicken or fish dishes and widely used in omelettes, risotto recipes, delicious soups, and sauces as well (Kozarski et al. 2015). *C. cibarius* has apricot‐like, cooked carrot aromatic odor which is due to the presence of fatty aldehyde (octanal and (E)‐2‐octenal), ketone (3‐octanone), and alcoholic compounds ((E)‐2‐ octen‐1‐ol) (Aisala et al. 2019). *C. cibarius* contain taste modifier octadecadien‐12‐ynoic acids responsible for kokumi taste and several C18‐acetylenic acids (Mittermeier, Dunkel, and Hofmann 2018). Fons et al. (2003) identified various volatiles compounds in five Chanterelles including *C. cibarius*, and the major constituent was C_8_ derivatives, that is, 88.6%. These volatile components are widely reported for their various mushrooms like odors like, benzaldehyde (almond odor), benzyl alcohol (sweet‐spicy odor) as well as (E)‐1, 3‐Octadiene along with 2, 4‐decadienal responsible for apricot and plum flavors respectively. Politowicz et al. (2017) analyzed fresh and dried sporocarps of *C. cibarius* and reported 39 volatile compounds. There were three most prevalent compounds in fresh chanterelle, that is, 1‐ octen‐3‐ol, 1‐hexanol, and 2‐octen‐1‐ol, which were responsible for their peculiar aroma.

In Europe, after Penny Buns (*Boletus edulis*), golden chanterelle (*C. cibarius*) is the second most collected wild edible mushrooms as 1,88,000 t with 1 billion Euros worth per year (Lovrić et al. 2020; Lovrić et al. 2021). From prehistoric times to the present, edible wild mushrooms (EWM) have been highly coveted and demanded as important sources of daily food, traditional medicines, and commercial profit (Azeem, Hakeem, and Ali 2020). In several countries, EWM recognized as sustainable “meat of poverty” or “forest meat,” which is not originated from animal source (Dimitrijevic et al. 2018). The meaty taste in mushrooms is due to the presence of 5′‐nucleotides, particularly 5’‐GMP and *C. cibarius* also contains all of them with varied concentrations (Muszyńska et al. 2016). The consumption of the chanterelle alone is estimated to be between 150, 000 and 200, 000 metric tonnes per year worldwide (Kumari, Reddy, and Upadhyay 2011).

## 7 Commercial Harvest of Cantharellus

Wild mushrooms have been grouped under the category of nontimber forest products (NTFPs), through which food and income resources can be generated (Boa 2004; De‐Roman and Boa 2006; Devkota 2008; Liu et al. 2018). In 2017, around 10.2 million tonnes of mushrooms and truffles were produced globally; where Asia accounted for 80.5% between 2013 and 2017; Europe contributed 13.2%; Africa as 0.3%; America as 5.5%; and Oceania contributed 0.5% (FAO STAT 2019). In Europe and North America, chanterelles are particularly regarded for their culinary value. They are among the most widely consumed edible wild mushrooms due to their graceful stature, delicate flavor, and fruity aroma (Arora 1990). Chanterelles are nutritious, having about 10% proteins by weight (Pilz et al. 2003), contain higher content of vitamin A, as well as the richest source of vitamin D found in nature.

The annual global production of chanterelles is estimated by Watling (1997), to be 1.67 billion USD, and the global trade of chanterelle is expanding. The estimated world's chanterelle trade to be around 2, 00, 000 metric tonnes, accounting for annual worth of approximately $1.25 to $1.4 billion (Hall et al. 1998; Hall and Yun 2000). Although prices paid to harvesters fluctuate daily and seasonally in the Pacific Northwest. The annual average prices were reported consistent by Blatner and Alexander (1998), which were $2.95/pound in 1992, $4.00 in 1994, $3.02 in 1995, and in 1996 it was $3.06/pound. Rowe (1997) reported the price per pound in 1992 ranged from $ 1.25 to $ 8.00 over the course of a season, with an average of $2.00.

European countries like Turkey, Bulgaria, and Serbia are at the top in the production of *C. cibarius* mushroom (Sumic et al. 2016). Since 1970s, *Cantharellus californicus* has been gathered for commercial purposes. For a very long time, harvesters have recognized it as a unique species and refer to it as the “mud chanterelle.” Many chefs outside of California show least interest in *C. californicus* than other chanterelles because of its large size, high fibrous, and nonaromatic nature. During commercial harvesting, harvesters in California and Pacific Northwest typically receive 1 to 5 USD per pound (2 to 11 kg^−1^) for *C. formosus* as compared to USD 4 to 10 pound^−1^ (9 to 22 kg^−1^), or higher price could be obtained for mud chanterelle if selling directly to markets and restaurants (Arora and Dunham 2008). According to an estimate, between 1,50,000 and 2,00,000 t of wild chanterelles are produced per annum, with market price of over $1.7 billion (Mitchell and Hobby 2010).

Europe is the main market for wild edible mushrooms, with France and Germany having the highest demand. In 1992, harvesters from Idaho, Oregon, and Washington were paid in total USD 20,267,080 for 3,935,254 pounds of wild edible mushrooms, where harvesters were paid USD 3,664,261 for 1,135,175 pounds of chanterelle (Schlosser and Blatner 1994). In British Columbia, the total quantity of chanterelles harvested varies from 187,500 kg in bad season to 750,000 kg in good season (Wills and Lipsey 1999). The largest production region in British Columbia is Haida Gwaii. Approximately 11,500 kg of total production is estimated to be produced there in a good year, with the net worth of pickers ranging from CAD $ 2,25,000 to $ 350,000 based on a price range of $5.50 to $9.25 kg^−1^ (Tedder, Mitchell, and Farran 2000). According to a study by Ehlers and Hobby (2010), in northern Vancouver's Island, pickers receive prices CAD$ 2.20 to $16.50 kg^−1^ for fresh chanterelles. However, commercial pickers claimed to harvest 4.6–27.3 kg per day with expected daily earning of $22.50 to $135.00, whereas some pickers claimed the collection of 45 kg per day, which at the best price bring the earnings of $ 750. The wild mushroom industry as a whole is governed by the level of variation in harvest amounts and prices paid.

## 8 Cultivation Practices

Mushroom cultivation could be an alternative strategy for the conservation of certain important and endangered plant species, wherein dependence on wild resources can be reduced for nutraceuticals and phyto‐chemicals for drug preparation. Over the past ten years, the production of mushrooms and truffles has climbed from 6.90 to 10.24 million metric tonnes (Ho, Zulkifli, and Tan 2020). In next few years, the annual value of the worldwide mushroom market anticipated to be surpassing US 50 billion dollars. An approximate 0.13 million mushrooms were produced in India between 2010 and 2017, representing an average and annual increase of 4.3% growth rate (Raman et al. 2018). It is predicted that by 2023, the value of the edible mushroom industry might reach USD 62.19 billion (Research and Markets 2021). Although there are other types of mushrooms, including mycorrhizal, saprophytic, and parasitic types, but usually saprophytic are selected for artificial cultivation (Stamets 2000). Around the world, 200 different types of mushrooms are consumed as superfoods (Kalac 2013), and only three edible mushroom species—*Agaricus bisporus*, *Pleurotus ostreatus*, and *Volvariella volvacea*—are generally grown, out of the 33 species that are currently being cultivated worldwide (Erbiai et al. 2021). More than 30‐fold production of cultivated edible mushrooms has been achieved, and per capita consumption has increased 4.7‐fold globally since 1978 (Royse, Baars, and Tan 2017; Beelman, Kalaras, and Richie 2019).

The first functional committee for mushroom cultivation was established in 1894 at the “Mushroom capital of the world,” that is, Pennsylvania (Beyer 2003). Mushroom requires special conditions for their growth and production, like low temperature, consistent humidity, light exposure, good aeration, and appropriate substrate composition (Wani, Bodha, and Wani 2012; Hou et al. 2017). The first success in commercially production of mushroom was achieved by a Frenchman in 1978, who cultured *Agaricus bisporus* underground in quarries nearby Paris (Niazi and Ghafoor 2021).

In 1996, for the first time, Danell achieved sporocarp production of *C. cibarius* under greenhouse condition. Commercial production of chanterelles in the glasshouse condition is quite difficult, but seedlings inoculated with selected mycelia may allow commercial production. Danell planted inoculated chanterelle ectomycorrhizal seedlings into pots in a greenhouse. After several months, when seedlings acquired 16 months of age and height of 0.5 m, he reported abundant chanterelle ectomycorrhizae and fruiting bodies from the drainage holes of the pots (Danell and Camacho 1997).

There are orchards for the production of black truffles (Giovannetti et al. 1994; Chevalier and Frochot 1997). A chanterelle orchard of 225 m^2^ can be established by planting 100 seedlings in a square at a distance of 1.5 m from each other. In Scottish forests, Slee (1991) reported a production of 50 kg *C. cibarius* ha^−1^ year^−1^, while in southern Sweden, an irrigated field plot (15 × 35 m) yielded a total of 17 kg FW of *C. cibarius* in 1992 (Danell 1994a). In 1994, Danell developed a rapid technique for the large‐scale production of chanterelles. His technique was totally based on methods of McLaughlin (1970) and Jentschke, Godbold, and Hütterman (1991). He inoculated the seedlings of *Pinus sylvestris* and *Picea abies* with *C. cibarius* hyphal suspension and achieved ECM formation in 8 weeks. Several sporocarps and primordia of *C. cibarius* were observed with 16‐month‐old *Pinus sylvestris* seedlings in pots.

## 9 Preservation and Processing Methods

Shelf life and postharvest quality of edible mushrooms could be improved by developing durable techniques whether for short‐ or long‐term preservation. There are several such preservation techniques that are lately being used include, physical, chemical, and thermal methods (Bulam, Ustun, and Peksen 2021). The edible mushrooms can get altered chemically during preservation and thus adversely affect their nutritional contents, organoleptic and bioactive qualities, and commercial importance (Xue et al. 2017; Thakur 2018; Marcal et al. 2021). The chanterelles, preserved in vinegar and olive oil, were observed to have a significant decrease in the quantity and quality of the bioactive compounds like phenolics and organic acids (Valentao et al. 2005). Additionally, it is to be remembering that deep‐freezing is a common practice for enhancing storage stability and enables year‐round mushroom consumption without seasonal constraints (Manzi et al. 2004).

Postharvest processing techniques are little explored for chanterelle mushrooms. The consumption of fruiting bodies of *C. cibarius* is safe and it can be taken as fresh, dried, frozen or in pickled forms. As compared to fresh, frozen, or canned mushrooms, the shelf life of dried mushrooms is much longer. To preserve the quality of the raw material, drying can be done using the vacuum drying process at lower temperatures and low oxygen level. Vacuum‐drying is an excellent method for materials that can alter or be damaged by the impact of high temperatures. The vacuum pressure of 10 kPa and 50°C temperature is ideal for reducing water activity, maximizing the total phenolic content and the ability of vacuum‐dried chanterelle mushrooms to rehydrate (Sumic et al. 2013, 2016).

Blanching assists in food preservation techniques by reducing the numbers of contaminating microorganisms on their surfaces (Fellows 2017). Blanching of fresh sliced *C. cibarius* using gently boiling potable water/distilled water or with mineral/deionized water for 5–15 min caused 15% leaching of mercury (Hg), while 35% for sliced and deep‐freezed fruiting bodies. Total leaching rate (i.e., 15 to 37%) of Hg was reported for fresh pickled *C. cibarius* and it was between 37% and 39% when deep‐frozen fruiting bodies were processed (Falandysz and Drewnowska 2017). Similarly, blanching fresh chanterelles reduces the amount of cadmium by 11%–36%, whereas approx. 40% for deep‐freezed mushrooms. Blanching the double picked chanterelles resulted in extra loss of Cd (37%–71%). When chanterelle sporocarps were blanched and then further pickled, the overall Cd leaching rate was between 77% and 91%. The blanching and pickling greatly reduce the amount of Cd in *C. cibarius* (Drewnowska et al. 2017).

In *C. cibarius* samples, convective predrying and vacuum microwave finish drying (CPD‐VMFD) at 70°C led to an increased content (136 g 100 g^−1^ DW) of volatile chemicals, but neither essential amino acids nor nonessential amino acids were observed to be altered (Politowicz et al. 2017). However, drying of mushroom slices (5–7 mm) in a tunnel dryer at 70°C for 4–5 h resulted in lower content of vitamin C and total anthocyanins, which might be due to the destruction by drying (Salihovic et al. 2021). In addition, the methods like lyophilization (at −40°C) and sun‐drying (25 ± 2°C) are more advantageous in increasing the quantities of bio‐elements (Zn, Cu, Fe, and Mg) in edible mushrooms than drying in a dryer (Kala et al. 2021).

Modified atmosphere packaging (MAP) method considerably reduces decay and weight loss of *C. cibarius* (Ozturk, Havsut, and Yıldız 2021). As storage period passage, the significant increase was reported in total phenolics, proteins, vitamin C, ash, and dry matter as well as in antioxidant activity. Preservation and processing of mushrooms with certain bacteria is also a unique method for maintaining quality and long‐term storage. Jabłonska‐Rys et al. (2016) performed lactic acid fermentation of *C. cibarius* with *Lactobacillus plantarum* strain and reported that this bacterium can effectively lower pH and enhance gallic and ferulic acids contents in fermented mushrooms.

Thermal processing also increased the release of bioelements like zinc (Muszyńska et al. 2016), Mg, Zn, Fe, and Cu from *C. cibarius* into artificial digestive juices (Kala et al. 2017, 2019, 2021).

## 10 Conclusion and Future Prospects

The review emphasizes the significance of *C. cibarius* as nutritional, nutraceutical and medicinal benefits on the health of consumers. In this review, conventional as well as novel preservation and processing techniques are discussed along with cultivation techniques. As we know, mushrooms have opened an outdoor in our daily diet schedule because of high nutritional, nutraceutical, and medicinal values. In addition, mushrooms have been regarded as nutraceutical foods that are effective in preventing diseases like cancer and other severe life‐threatening disorders like neurodegeneration, hypertension, diabetes, and hypercholesterolemia. Future study should be concentrated on the discovery of new effective drugs for the well‐being of mankind. There should be a technique for recovering some essential compounds from waste generated during production and processing which could be valorized by both food and pharmaceutical industries. Finally, the mechanisms of action of bioactive compounds found in mushrooms should be studied at cellular level.

## Author Contributions

**Ajay Kumar:** conceptualization (equal), visualization (lead), writing – original draft (equal), writing – review and editing (equal). **Reema Devi:** formal analysis (equal). **Rajni Dhalaria:** data curation (equal), writing – review and editing (equal). **Ashwani Tapwal:** data curation (equal), writing – review and editing (equal). **Rachna Verma:** data curation (equal), writing – review and editing (equal). **Summya Rashid:** data curation (equal), writing – review and editing (supporting). **Gehan M. Elossaily:** data curation (equal), writing – review and editing (supporting). **Khalid Ali Khan:** data curation (equal), writing – review and editing (supporting). **Kow‐Tong Chen:** data curation (supporting), writing – review and editing (equal). **Tarun Verma:** data curation (equal), supervision (equal), writing – review and editing (equal).

## Conflicts of Interest

The authors declare no conflicts of interest.

## Data Availability

The data generated or analyzed that support the findings of this study are available and included in this published article.
